# Systematic Study

**Published:** 1886-09

**Authors:** J. D. Moody

**Affiliations:** Mendota, Ill.


### SYSTEMATIC STUDY.

BY J. D. MOODY, D. D. S., MENDOTA, ILL.

In the editorial entitled "Post-Graduate Study," in the PRACTITIONER for June, you suggest "courses of lectures on definite themes somewhat after the manner of the Chautauqua Literary Course." I desire to call attention to a plan of study which, until something better is provided for us, will offer to the dental student a well arranged course of scientific work. While not exactly meeting the needs of a dental student, yet, in the absence of any course especially prepared for him, it will meet the want very fully, and his work will not have been thrown away, should he at any future time desire to take up a course especially prepared for him. By dental student I mean one who has an inclination to study and who makes an attempt to pursue it, whether preparing for college or engaged in practice.

The plan of which I speak is a course of study arranged by the authorities of the Chautauqua University, to be carried on by correspondence. It is a part of the regular curriculum of the School of Biology, to be taken up and pursued as special work, independent of the other branches in that course. With the aid of a fair microscope and the directions of the tutor in charge, a great deal of pleasure and profit can be obtained from it, and it will greatly enlarge the mental horizon. He who follows it will be laying foundations of permanent value upon which future work can be built. It will give a better understanding of much with which we have to do, and tend towards a real satisfaction with ourselves.

I know whereof I speak, having just finished the first year's work myself. It is arranged to occupy four years, but the student is not limited as to time. He can go faster or slower as he finds himself best able to do.

The course is as follows:

| | |
|---|---|
| First Course: | Elementary Biology.<br>Dissection of Fish.<br>Dissection of Chelonian.<br>Dissection of Bird.<br>Dissection of Rat.<br>Botany. |
| Second Course: | Osteology { Human. Comparative.<br>Histology.<br>Physiology. |
| Third Course: | Botany.<br>Comparative Anatomy.<br>Embryology. |
| Fourth Course: | Animal Life.<br>Origin of Cultivated Plants.<br>The Geographical Distribution of Animals and Plants. |

The syllabus of the first course is as follows:

(*A*) Plants.

| | | | |
|---|---|---|---|
| Yeast. | Chara. | Mucor. | Bean Plant. |
| Penicillium. | Fern. | Spirogyra. | Indian Corn. |
| Protococcus. | Bacteria. | Nitella. | |

(*B*) Animals.

| | | | |
|---|---|---|---|
| Amœba. | Cray Fish. | Star Fish. | Frog. |
| Hydra. | Perch. | Earth Worm. | Pigeon. |
| Sea Urchin. | Terrapin. | Oyster. | Rat. |
| Mussel. | Sponge. | Grasshopper. | |

(*C*) Botany.

Elements of Systematic Botany and Plant Analysis.

This work will be found intensely interesting to any one who will take it up. Full particulars can be had by addressing R. S. Holmes, Registrar, Plainfield, New Jersey. The school year begins in October, and ends in June.

I hope that the day is not far distant when some similar plan, modified to suit the needs of the dentist, will be arranged for us by some competent authority.

